# Negative Example Selection for Protein Function Prediction: The NoGO Database

**DOI:** 10.1371/journal.pcbi.1003644

**Published:** 2014-06-12

**Authors:** Noah Youngs, Duncan Penfold-Brown, Richard Bonneau, Dennis Shasha

**Affiliations:** 1 Department of Computer Science, New York University, New York, New York, United States of America; 2 Social Media and Political Participation Lab, New York University, New York, New York, United States of America; 3 Department of Biology, New York University, New York, New York, United States of America; 4 Center for Genomics and Systems Biology, Department of Biology, New York University, New York, New York, United States of America; Indiana University, United States of America

## Abstract

Negative examples – genes that are known *not* to carry out a given protein function – are rarely recorded in genome and proteome annotation databases, such as the Gene Ontology database. Negative examples are required, however, for several of the most powerful machine learning methods for integrative protein function prediction. Most protein function prediction efforts have relied on a variety of heuristics for the choice of negative examples. Determining the accuracy of methods for negative example prediction is itself a non-trivial task, given that the Open World Assumption as applied to gene annotations rules out many traditional validation metrics. We present a rigorous comparison of these heuristics, utilizing a temporal holdout, and a novel evaluation strategy for negative examples. We add to this comparison several algorithms adapted from Positive-Unlabeled learning scenarios in text-classification, which are the current state of the art methods for generating negative examples in low-density annotation contexts. Lastly, we present two novel algorithms of our own construction, one based on empirical conditional probability, and the other using topic modeling applied to genes and annotations. We demonstrate that our algorithms achieve significantly fewer incorrect negative example predictions than the current state of the art, using multiple benchmarks covering multiple organisms. Our methods may be applied to generate negative examples for any type of method that deals with protein function, and to this end we provide a database of negative examples in several well-studied organisms, for general use (The NoGO database, available at: bonneaulab.bio.nyu.edu/nogo.html).

## Author Summary

Many machine learning methods have been applied to the task of predicting the biological function of proteins based on a variety of available data. The majority of these methods require negative examples: proteins that are known *not* to perform a function, in order to achieve meaningful predictions, but negative examples are often not available. In addition, past heuristic methods for negative example selection suffer from a high error rate. Here, we rigorously compare two novel algorithms against past heuristics, as well as some algorithms adapted from a similar task in text-classification. Through this comparison, performed on several different benchmarks, we demonstrate that our algorithms make significantly fewer mistakes when predicting negative examples. We also provide a database of negative examples for general use in machine learning for protein function prediction (The NoGO database, available at: bonneaulab.bio.nyu.edu/nogo.html).

## Introduction

Despite the recent influx of machine learning algorithms applied to function prediction, there has been relatively little study devoted to the issue of class imbalance in function labels. This imbalance stems from the fact that the current standard set of labels for protein functions, the Gene Ontology (GO) database [1], rarely stores which proteins do *not* possess a function. If no annotation is present for a given gene to a particular GO term, it does not mean that such a gene is a negative example for that term, but rather that it is *either* a negative example or a positive example that has yet to be annotated. This situation arises due to experimental constraints: function assays are typically applied to single proteins and that protein function can be context dependent, making negative statements/labels quite uncertain, and leading to very few (or for most protein functions, not any) verified negative examples. This imbalance presents an obvious problem for the vast majority of machine learning techniques, which require enough examples of both the positive and negative class in order to train an accurate predictor. Without these labeled negative examples, authors often resort to heuristics in order to define the non-positive class; but mistakes stemming from these heuristics can lead to false negatives in the training set, and are detrimental to classifier performance.

The situation described above, in which the only known labels are of the positive class, is not unique to the protein function prediction (PFP) problem, but also occurs in several other domains. It has been given the name Positive-Unlabeled (PU) learning, and there has been a surge of interest lately in this particular subset of semi-supervised machine learning problems. One branch of PU algorithms attempts to learn in a one-class scenario, as has been applied to biology, specifically mRNA detection [2]. As the authors point out, however, 2-class machine algorithms often perform better when the negative class can be well defined. In another 1-step algorithm [3], the authors demonstrate that if certain conditions hold, learning without explicitly knowing negative examples is possible and even more accurate than existing methods. Unfortunately, this assumption requires the probability of a true positive example being labeled to be independent of the example itself (the set of observed positive labels should be selected at random from the total set of true positives). Since GO terms are often propagated via homology methods, there is a high degree of correlation between many of the labeled positive examples, and so this assumption does not hold in our domain. Thus we focus on the majority of PU algorithms, which proceed by first predicting a set of reliable negative examples before applying a traditional machine learning classifier to the enriched data as usual. These 2-step algorithms take many forms (see [4] for review of these methods), but in this work we will refer to two main subcategories: passive 2-step PU algorithms, which learn the negative examples through a separate mechanism from the classifying algorithm, and active 2-step PU algorithms, which work in conjunction with the classifier to learn the negative examples.

The main focus of PU-learning literature has been to improve text classification [4], a problem in which labeling a document's topics is time-intensive, and it is not practical to label all the topics a document does not contain. Yet the analogies to protein function are clear: proteins are rarely labeled with the functions they do NOT possess, and proteins are nearly always multi-topic, in that the annotation of a protein to a particular GO-term does not exclude the potential for several other functional classifications (we use the word “function” synonymously with “GO term”, regardless of which branch of GO that term occurs in). Therefore PU algorithms are applicable to the function prediction problem, and hold great potential for improvements in machine learning algorithms applied in this context. For example, we have previously shown that more-reliable negative examples boost the predictive power of protein function prediction algorithms [5].

We proceed by focusing directly on the first step of the PU learning task, namely generating a reliable set of negative examples for protein function and directly evaluating the quality of our negative examples, rather than their indirect effect on classifier performance. While PU learning has been applied to the biological domain before [2], [6], [7], to the best of our knowledge no study has focused on evaluating the quality of negative examples for GO functions. We examine many of the heuristics used for protein function negative examples in the past, including: designating all genes that don't have a particular label as being negative for that label [8], randomly sampling genes and assuming the probability of getting a false negative is low (often done when predicting protein-protein interactions, as in [9]), and using genes with annotations in sibling categories of the category of interest as negative examples [10], [11]. To these heuristics we add two common PU algorithms used in text classification but here adapted to PFP, the Rocchio algorithm [12] and the “1-DNF” algorithm [13], as well as our ALBNeg algorithm [5], and one of the few previously-published protein-negative-example-selection algorithms, the AGPS algorithm [7]. In addition, we present two new techniques: the first, Selection of Negatives through Observed Bias (SNOB), is an extension of our ALBNeg algorithm (which can itself be viewed as a generalization of the “1-DNF” PU algorithm), while the second, Negative Examples from Topic Likelihood (NETL), is based on a Latent Dirichlet Topic model of GO data.

Our algorithms, as well as competing algorithms borrowed from text classification, require only existing GO annotations in order to predict negative examples. As new annotations are continuously added to GO this allows testing via training on archived GO data, and examining the number of incorrectly predicted negative examples using current GO data to identify true positives that were predicted to be negative. The AGPS method utilizes additional feature data, such as Gene Expression, Protein-Protein-Interaction, etc., but can still be evaluated in the same manner as the other algorithms. We provide a case study to show how these examples can benefit the performance of other algorithms, specifically a function prediction method tested in *A. thaliana* [14]. Additionally, we demonstrate increases in function prediction accuracy when our negative examples are used, testing on human, mouse, and yeast proteins, using our earlier-published function prediction algorithm [5]. Lastly, we provide a resource, NoGO, which contains lists of high-quality negative examples for GO categories in a variety of well-studied organisms (Human, Mouse, Worm, Yeast, Rice, and Arabidopsis).

## Results

### Evaluation of Negative Example Quality

Function prediction results are biased negatively (estimations of function prediction accuracy are typically lower limits) by the fact that a positive prediction without a corresponding validation annotation might simply indicate lack of study of the gene rather than an incorrect prediction. It therefore follows that negative example validations are biased by the same effect, but positively (estimated error rates are lower bounds). Just because a gene is not annotated with the function in the validation data doesn't guarantee that it was correctly identified as a negative example. In order to attempt to rigorously evaluate potential negative example selection algorithms, we utilize the average number of false negative predictions over categories in each of the three branches of GO.

We determine false negatives through a temporal holdout in order to mitigate bias [15], running all of our algorithms on data from the human genome obtained in Oct. 2010, and then validating with data obtained in Oct. 2012. This process involves restricting the training phase of all algorithms to data available in Oct. 2010, removing the potential for test and training data correlation that can happen during cross-validation. Any gene that was predicted as a negative example from 2010 data, which received a positive annotation in the 2012 data, is considered an error in prediction (a false negative example). For extra stringency, we consider an “Inferred by Electronic Annotation” (IEA) evidence code annotation as an indication of false negativity (even though these types of annotations are traditionally considered less reliable). For completeness, we also include an evaluation without considering IEA annotations, presented in Figures S4 and S5.

Prediction errors are calculated separately for each GO term, and then averaged together within each branch of GO. Only categories that have between 3 and 300 annotations are evaluated, so as to consider only terms specific enough to be interesting but not so specific as to have little chance of being validated, since prediction errors can be observed only if new annotations appear for the category in question in the Oct 2012 data that were not present in the Oct 2010 data.

Additionally we focus on a specific GO term in human (RNA Binding), augmenting the temporal validation with annotations from a recent high throughput screen for RNA binding proteins [16]. Lastly, we evaluate using a gold-standard set for a single GO term in the yeast genome [17].

As the trivial solution (predicting no negative examples) would obviously have the lowest number of false negatives, we present results in two dimensions, where the vertical axis is average number of false negatives, and the horizontal axis is number of negative examples predicted (in this setup, the origin represents the trivial solution, while the upper right corner of the plot represent choosing all non-positive genes as negatives). Algorithms that do not have the capability to vary the number of negative examples that they predict appear as points on the performance graph, instead of lines. Because prediction errors can be evaluated only if new annotations appear during the course of the temporal holdout time period, the error rate calculated is an observed error rate, rather than the true error rate. This observed rate will vary in magnitude from GO term to GO term, as it is bounded from above by the number of new annotations. Since the magnitude of the number of false negatives in each branch is dependent on the total number of new annotations added in that branch between 2010 and 2012, the numbers cannot be compared across branches. In order to provide a reference point that is comparable across each branch, we treat the performance of random selection of negative examples as a baseline. Thus while the magnitude of the observed error rate cannot be compared across branches, the difference between an algorithm and the random baseline is comparable, both across branches and between GO-terms of differing specificity.

### SNOB and NETL, Two New Novel Negative Selection Algorithms

Our first novel negative example selection algorithm, Selection of Negatives through Observed Bias (SNOB), is an extension of our previously published ALBNeg algorithm [5], which selected negative examples for a function based on whether or not a gene's most specific functional annotations had ever appeared alongside that function. ALBNeg in turn can be viewed as a generalization of a popular passive 2-step PU-learning algorithm known as “1-DNF” negative example selection. This algorithm works in the context of text classification by identifying words that are enriched among the positive class, and using as negatives all unlabeled documents that do not contain any of these positive “indicator” words [4]. We consider each GO term annotation as a “word” in the “document” of a protein, then apply the “1-DNF” technique to choose negative examples for a protein function by excluding proteins with GO terms that are enriched among proteins containing the function of interest.

In ALBNeg, we generalized the idea of “enrichment”, by computing the empirical conditional probability of the GO function of interest, denoted *g*, given the presence of each other GO function in all three branches [5]. Proteins whose most specific annotations had non-zero conditional probabilities of appearing in a gene alongside *g* were ruled out from the potential negative set for *g*, effectively using the conditional probability as an indicator of potential positivity in the same way that the “1-DNF” algorithm uses enriched terms.

In our new algorithm (SNOB), presented here, we follow the same approach as ALBNeg, and for each GO term *g*, compute the pairwise empirical conditional probability of seeing *g* given the presence of each other GO term. We further develop ALBNeg, i) by including IEA annotations in our calculations as well. We then obtain a score for each protein for each GO term *g*, by averaging the conditional probabilities of all GO terms (including IEA) annotated to that protein, ii) by including all GO terms in the average, not just the most specific terms, and iii) instead of choosing all proteins with a score of 0 as negatives for the function *g*, we allow the user to set a desired number *n* of negative examples, and choose the *n* proteins with the lowest scores as our negatives for *g*. See the Methods section for details of this calculation.

Our second novel algorithm, Negative Examples from Topic Likelihood (NETL), again treats proteins analogously to “documents”, with the GO terms annotated to each protein serving analogously to a document's “words”, but now we consider the proteins to have latent “topics” as well. These hidden topics represent the “true” function of the protein, both accounting for new functions (functions not annotated because they have to be verified/tested) as well as errors and missannotations (having a GO annotation does not guarantee that a protein actually performs the function in question due to potential errors in annotation, especially with IEA annotations). We can then apply a multi-topic inference algorithm, specifically Latent Dirichlet Allocation [18], to learn the distribution of these latent topics, or “true” functions, and also learn the conditional distribution of the “words” or annotated GO terms based on those topics. Once these distributions are known, NETL selects as negatives the proteins whose latent topic distributions are as dissimilar from the positive class as possible, allowing the user to specify how many negative examples are desired.

Ideally each latent topic would represent a single GO term, but since the size of the vocabulary in our corpus is also equal to the number of GO terms, this is not feasible. Instead, we utilize the GO hierarchy to select fewer but more general topics, while ensuring coverage of the entire GO tree. Such a setup does not guarantee an intuitively interpretable relation between the latent topics and specific GO terms: topic x does not directly correlate to any one GO term, but rather is likely a combination of GO terms. Thus the calculation of the likelihood of a particular protein being a negative example for a particular GO term is infeasible, and must instead be inferred through a similarity metric (see methods).

### Previous Methods for Negative Example Prediction

In order to provide a reference for the quality of our algorithm's negative examples, we include past heuristics used for negative example selection, as well as the popular passive 2-step PU algorithms, “1-DNF” and “Rocchio”, which we have adapted to the PFP context through the GO term “word” and protein “document” mechanism described above. In the case of the Rocchio algorithm, we made an additional adjustment allowing the number of negative examples to be varied (See Methods for details). We have chosen to focus on passive 2-step PU algorithms as the performance of active 2-step methods is intertwined with the performance of the underlying classification algorithm, as well as the input feature data. A stronger classifier will produce better negative examples, as will a classifier that can use more discriminative data. This increases the difficulty of judging the relative performance of active 2-step PU algorithms, as different classifiers utilize different mechanisms and datasets. These underlying differences make it difficult to correctly attribute relative performance of negative example selection to the 2-step algorithm itself, as opposed to the quality of the classifier or underlying data. Additionally, 2-step algorithms are self-reinforcing, in that the classifier identifies as negatives those proteins which are most different from the positive class by whatever mechanism that classifier is using, which only reinforces that particular kind of discrimination when the classifier is run again with the negative examples in the second step. In general, a classifier is better served with negative examples that are actually *more* similar under the classifying mechanism, in order to force the classifier to be more discriminative. Lastly, the passive 2-step algorithms presented here function solely with GO data input, allowing for very rapid calculations and avoiding the need to gather large amounts of feature data, which can quickly become difficult for less-studied organisms.

The exception to our focus on passive 2-step algorithms is the AGPS algorithm, which is an active 2-step PU algorithm with which we make a comparison. We have included this algorithm, as it is one of the few explicit negative example selection algorithms in the protein function prediction (PFP) literature.

### Performance of Negative Example Methods in *Homo sapiens*

**Figure 1:**
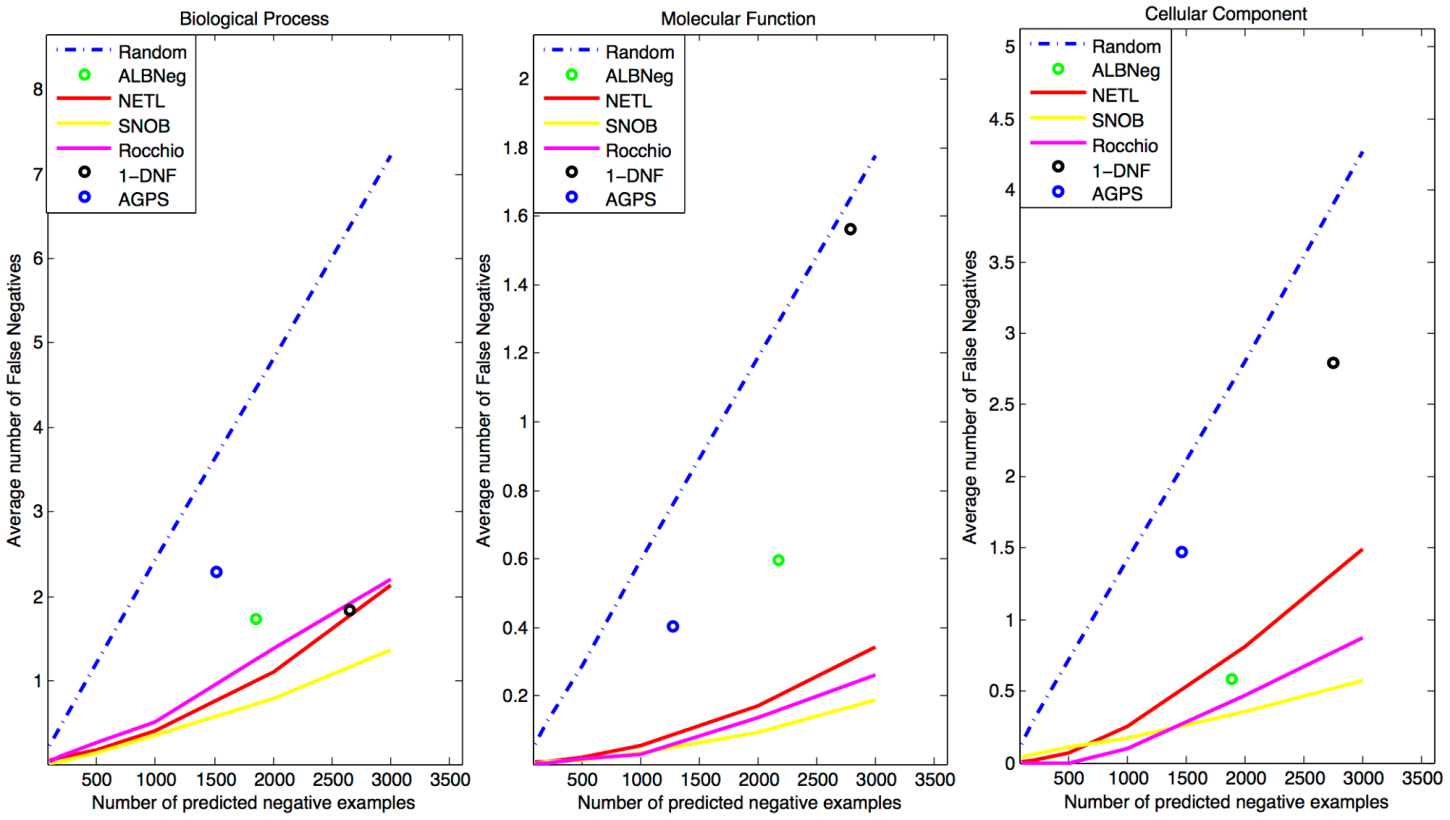
Performance measures for negative example prediction on the human genome. The number of erroneous negative example predictions is plotted as a function of the number of negative examples chosen, for each of the three branches of GO. The Rocchio, NETL, and SNOB algorithms show consistently strong performance, with SNOB achieving the lowest error rate in each branch. The “Sibling” and “All non-positive as negative” heuristics have been omitted, as their poor performance dramatically skewed the scale of the images (see figure S3 for results including the sibling method).

Results for the methods tested on the human proteome are presented in Figure 1. Among the methods tested, all algorithms performed better than the random baseline, with the exception of the sibling algorithm, whose weakness is also confirmed in [10]. The heuristic of choosing all non-positive genes as negative also does not perform better than the baseline, as it is itself a special case of the baseline where the number of negative examples is allowed to be the size of the genome (minus the number of positive examples). The best performance was achieved by the SNOB algorithm, which achieved an equal or lower average number of false negatives than all other algorithms, heuristics, and the baseline, across all three branches. The NETL algorithm, as well as our adaptation of the Rocchio algorithm to PFP, also exhibited strong performance compared with other algorithms.

Driving the performance of SNOB was its ability to achieve significantly fewer false negative predictions for more general GO categories (categories with more annotations in the human genome). Figure S1 shows false negative rates broken down by the specificity of the function, demonstrating that while the Rocchio algorithm can compete with or even outperform our SNOB algorithm on the most specific categories, it is eclipsed by SNOB in the more general ones. This discrepancy among categories is most likely driven by the fact that the SNOB algorithm directly utilizes the co-occurrence of functions (See the Methods section), and thus has less information to work with for the most specific categories.

While not performing as well as SNOB, our previously published ALBNeg algorithm still achieves comparable or better performance than the AGPS algorithm. This comes as somewhat of a surprise, as AGPS has the benefit of access to a wealth of biological data beyond the GO information utilized by our algorithms, and much of that data post-dates the training GO annotations, providing unfair bias due to the correlation of many data types with GO annotations. However, with that additional data comes additional noise, and we recognize that the AGPS algorithm might be able to improve upon its performance with additional parameter tuning and feature selection among the data inputs.

**Figure 2:**
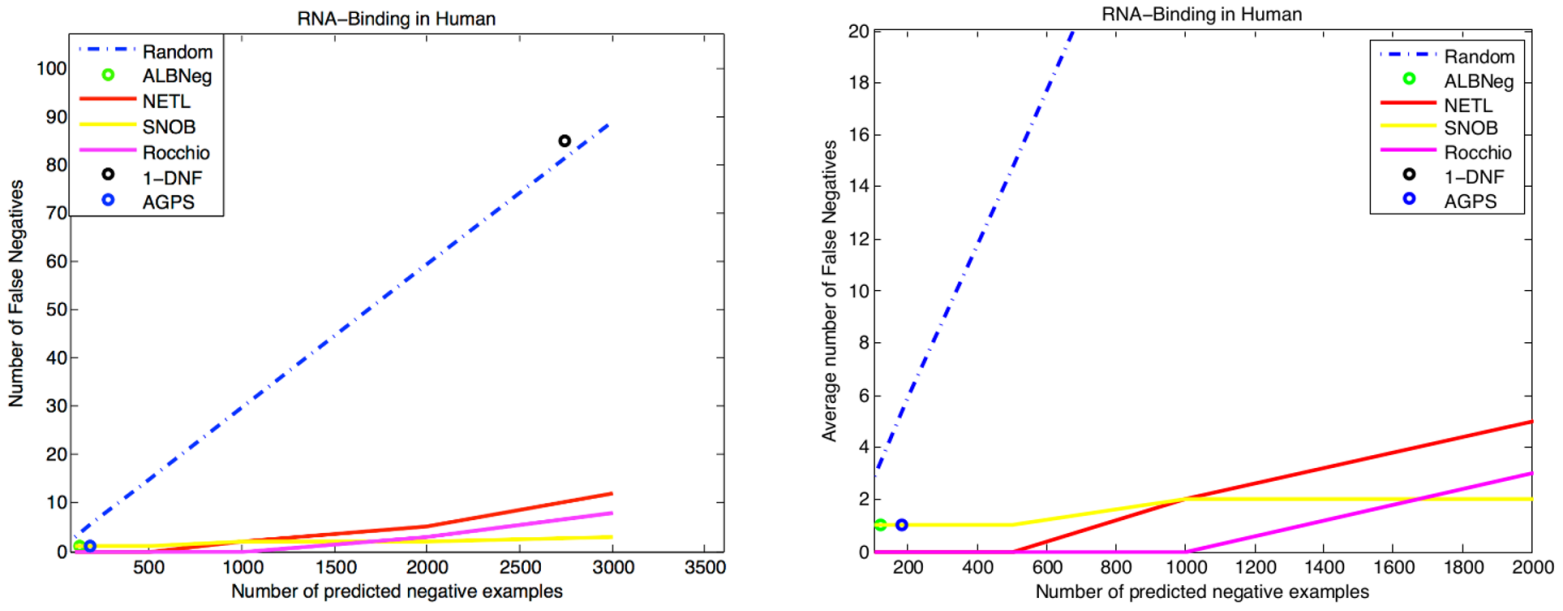
Performance measures for RNA binding. Performance of the competing algorithms on a specific GO category: GO:0003723 RNA binding, with validation data augmented by annotations taken from [16]. The left panel shows the complete results, while the right is a scaled to see the differences between algorithms near the origin. The SNOB algorithm achieves the fewest false negatives for large numbers of negative examples, while the Rocchio and NETL algorithms maintain a zero false negative rate for a greater number of negative examples.

The results presented in Figure 1 represent the average of a large number of individual evaluations, each with an error rate whose magnitude can vary largely depending upon the specificity of the term. We encourage the reader to examine Figure S1, which presents the same results but broken down by specificity, reducing the information lost by averaging. These results agree with those in Figure 1. To further substantiate our evaluation, we focused on one particular molecular function term: GO:0003723 RNA Binding, presented in Figure 2. We augmented the temporal holdout validation data with additional annotation not yet present in GO, but which have been experimentally verified in [16] via a large-scale genomics experiment designed to detect mRNA binding proteins genome-wide. These additional annotations significantly increase the number of potential false negative examples, allowing for greater discrimination between algorithms. Continuing in the same patterns as the entire human genome evaluation, the NETL, SNOB, and Rocchio algorithms perform similarly, and significantly better than the random baseline, with SNOB edging out the other two algorithms for larger numbers of negative predictions. Both NETL and Rocchio, however, maintain a zero false negative rate for a larger number of predicted negative examples than SNOB. AGPS and ALBNeg do well, but only provide a small number of negative examples, and both predict one false negative while NETL and Rocchio achieve zero errors at the same number of negative examples. The “1-DNF” algorithm performs very poorly on this category.

### Golden Set Evaluation in *S. cerevisiae*: Mitochondrial Organisation

In order to further explore the potential biases in the evaluation of negative example selection methods, we include evaluation on a gold-standard set of annotations in yeast, obtained from [17]. This golden set, for the biological process term GO:0007005 Mitochondrial Organization, represents an exhaustively verified set of annotations, such that all positive and negative occurrences of this GO term are known across the entire yeast genome. Because the number of true positives and negatives is known, this GO term in yeast allows us to utilize cross-validation on the data to calculate a Receiver-Operator Characteristic (ROC) curve or point for each algorithm. While cross-validation is problematic in the evaluation of function-prediction in general, due to the interconnectedness of GO and many types of feature data which introduces large positive bias into the evaluation, here we are examining and holding out only GO terms, and so such bias is mitigated.

**Figure 3:**
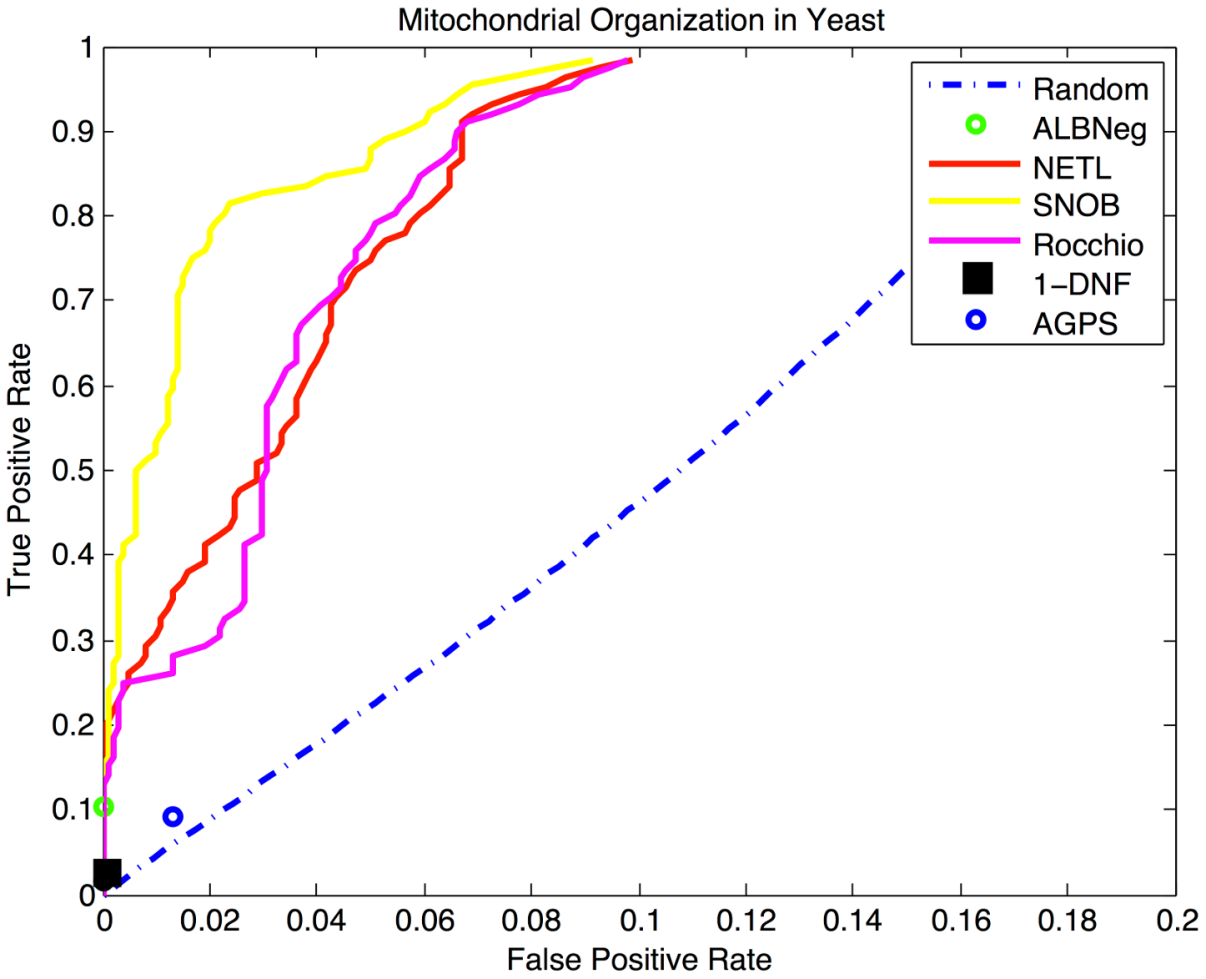
Performance measures for mitochondrian organization. ROC curves are depicted for each algorithm on the golden set of annotations for GO:0007005 in yeast, calculated through cross-validation. SNOB shows the highest area under the curve (AUC), followed by NETL and Rocchio, which have approximately equal AUCs.

In the yeast golden set, we see similar results (presented in Figure 3) as in our evaluation with human data: The SNOB algorithm is the strongest performer, followed closely by the Rocchio and NETL algorithms. The ALBNeg algorithm also performs well, achieving zero false assertions of negative functionality with a large number of predicted negative examples (473.2 on average). The 1-DNF algorithm also achieves zero false assertions of negative functionality, but with fewer predicted negative examples (only 76.6 on average), and the AGPS method predicts fewer negative examples than ALBNeg, with a much higher number of false negatives (2.6 on average). It is also worth noting that 59 of the 4625 negative examples in the golden set had received positive annotations for GO:0007005 in the years since the golden set was formed (the annotations set is updated accordingly here).

### Case Study: Improving Function Prediction in Human, Mouse, and Yeast

**Figure 4:**
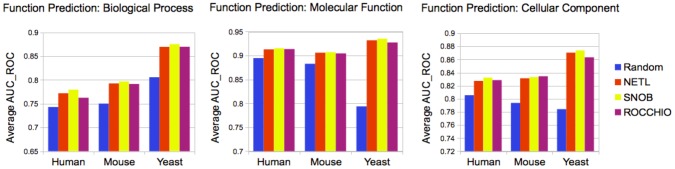
Performance measures for function prediction. AUC_ROC measures for function prediction using the best-performing negative example selection methods, with the random negative example selector included for comparison. Performance measures are broken up by ontology branch, and represent the average AUC_ROC for all GO terms predicted in that branch.

In order to demonstrate the importance of high quality negative examples, we use our previously published algorithm [5] to predict functions across all three branches of GO, for human, mouse, and yeast proteins. We validate these predictions with a temporal holdout (see methods), which enables us to compute the area under the curve (AUC) for the Receiver-Operator-Characteristic (ROC) plot. We repeat this process using negative examples selected by each of the best-performing negative-example-selection methods, as well as with random negative examples to serve as a baseline. Results are presented in Figure 4.

Comparing the average AUC_ROC values of function prediction with the negative examples selected by each method, we see relative performance very similar to our earlier evaluation of negative example quality. All three of the negative-example-selection algorithms yield much stronger function prediction performance than when negative examples are selected randomly from proteins lacking the positive example. Between the three algorithms, performance is fairly similar, with function prediction utilizing the SNOB negative examples slightly outperforming the other methods.

### Case Study: Improving Function Prediction in *Arabidopsis thaliana*

We apply our SNOB algorithm to the work of Puelma et al. [14], which employs discriminative local subspaces in gene expression networks to predict function in *Arabidopsis thaliana*. We choose this work as a case study because the authors specifically mention the importance of negative examples in their work, and devise an algorithmic approach for selecting high-confidence negative examples for the 101 biological process categories they used to test their PFP method. We use their provided data to select negative examples with SNOB, generating the same number of negative examples per category as the author's original algorithm (a total of 313592 across all categories). Table 1 shows the results of our case study, demonstrating that even though our algorithm only had access to 1/3 of the data it usually requires (here the authors provided only Biological Process data, and no data from the other two branches of GO), SNOB produces significantly fewer false negatives, negative examples with greater specificity, and performs better when evaluated by the metric chosen by the authors. It is also interesting to note that even though the rate of false negatives is very small (originally only 0.6%), further reduction still produces performance gains in downstream function prediction.

**Table 1:** Results of our SNOB algorithm vs. the algorithm published in [14].

Algorithm	False Negatives	Negative Frequency	Avg Enrichment P-Value
Puelma Neg	1806	71.88	39.00%
SNOB	1241	29.05	36.26%

The “False Negatives” column shows the total number of false negatives produced by each algorithm across all 101 BP categories examined in the paper, as determined by BP data collected by the authors two years after the training data. The “Negative Frequency” column shows the average number of times any gene was selected as a negative example for different function categories, if it was selected at all (a higher number means the same proteins are selected as negative examples across more categories). The “Avg Enrichment P-Value” column is the metric the authors used to evaluate their function predictions, with a lower value indicating better performance (see [14] for details).

### Negative GO (NoGO) Database

**Figure 5:**
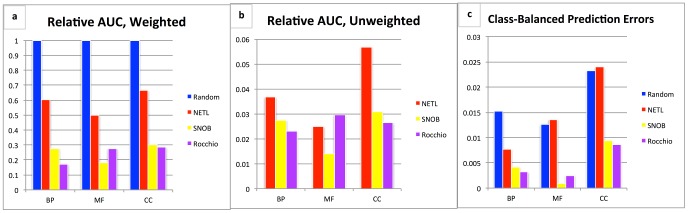
NoGO database performance statistics. Performance metrics for each algorithm in the NoGO database, averaged across all species, separated by branch of the GO ontology. A) The average area under the false negative curve, as a percentage of the area under the random baseline curve, weighted by the number of annotations in each GO category. B) The same values re-calculated so that each GO category contributes equally to the average, regardless of specificity (depicted without the random baseline as that is still 1.0 for every category but skews the scale of the plot). C) The false negative rate for each algorithm when predicting the same number of negative examples as the number of positive annotations for each GO category. Here lower numbers represent fewer errors.

We have collected negative example predictions from the SNOB, NETL, and Rocchio algorithms in an online database for use by other researchers. While the NoGO database uses the most current annotations for its ranking of negative examples, we have also included false negative rates for each species in the database, obtained from temporal holdouts on older data, to allow researchers to have a reference for the quality of negative examples in that organism. We describe the quality by the area under the false negative curve, as a percentage of the area under the random baseline curve, allowing the number of negative examples to range up to 20% of the size of the genome of that organism. Results are presented in Figure 5.

SNOB and Rocchio achieve the lowest overall errors, with the performance gap between Rocchio and NETL larger than in our other evaluations (see figure S2 for performance broken down by organism). The reduction of the performance gap between NETL and Rocchio in Figure 5b as compared to Figure 5a, indicates that while Rocchio performs better on more general categories, NETL's performance is on par with or better than Rocchio for the more specific GO terms (and thus a greater number of GO terms). It is also interesting to note that across all organisms, SNOB and Rocchio perform similarly on cellular component terms, SNOB has stronger performance on molecular function terms, and Rocchio performs better on biological process terms, suggesting systematic differences in the way that GO annotations relate to each other within each of the three branches.

Our Web interface to the NoGO database provides a plot for each GO function that shows the number of false negative predictions as a function of the number of negative examples chosen (Figure 2 is an example of such a plot, for GO:0003723). This allows researchers to make an informed decision about which algorithm to use for their specific organism, GO terms, and task. These plots also allow researchers to determine how many negative examples to use for each category (see methods).

## Discussion

We have demonstrated (using the human, yeast, and *A. Thaliana* proteomes) that the SNOB algorithm achieves significantly lower prediction errors when predicting negative examples than several previously described alternative approaches (including heuristics, techniques borrowed from PU-learning in text classification, and other negative-example prediction algorithms). These results, supported by additional literature that has explored the inter-relationships between GO categories [19], [20], indicate that despite lacking a significant number of negative annotations, the GO database encodes implicit information about likely negative examples via its positive annotations. Additionally, these pairwise term implications span all three branches of GO (cellular component, biological processes and molecular function).

Despite the success of our approach, there will inevitably be cases where the information from GO alone is not enough to predict a good set of negative examples. So-called “moonlighting” proteins, for example, can have unique combinations of functions that defy conventional annotation patterns. Additionally, approaches that rely on existing GO annotations are limited to proteins that have already been studied to some extent, which in many organisms can be a relatively small proportion of the genome. For these reasons, our group is considering active methods that can incorporate additional data types (such as gene expression, protein-protein interaction, domain structure, etc.).

The algorithms presented here represent a significant improvement over the active 2-step AGPS method that has access to data outside of GO. Our SNOB algorithm achieved a lower false negative rate than any other comparison algorithm tested, significantly lower than the “1-DNF” algorithm that served as its conceptual basis. Through our case study in Arabidopsis, SNOB also demonstrated its ability to improve existing function prediction algorithms. Youngs et al. 2013 [5] showed that even a moderate increase in the quality of negative examples has the power to improve function prediction in general, and those results are replicated here by our case study in human, mouse, and yeast. We have shown the ability of high quality negative examples to improve function prediction accuracy, again with the SNOB algorithm achieving the best results. Additionally, this case study represents a very basic use of these negative example methods, and we believe even further accuracy can be gained by more careful selection of the number of negative examples chosen for each prediction task.

Further work includes the incorporation of additional data types, and potentially the use of active 2-step PU methods. Another potentially fruitful avenue is the explicit incorporation of the GO hierarchy in a negative example method. While GO annotations obey the “true path rule”, meaning that every protein with an annotation *a* also implicitly has all annotations which are ancestors of *a*, negative annotations follow the inverse of this rule: a protein *p* that is a negative for *g* is also implicitly a negative for all descendants of *a*. This rule holds for the molecular function branch of GO, but is more complex in the biological process and cellular component branches, as there is more than one type of ancestry (terms may be direct descents, or connected by a “part-of” link, for example). These differences most likely account for some of the systematic performance differences of different algorithms on each branch of GO across all the organisms in the NoGO database.

These systematic performance differences across branches, combined with the fact that our GO-term specificity effects algorithms' relative performance, suggest the potential utility of ensemble methods (a combination of methods that use one of multiple algorithms depending on a GO term's specificity, placement in the tree, and desired size of the negative class). It is quite natural to think that the optimal algorithm will be quite different for predicting rare functions (functions that with only a handful of examples of per genome) and common functions (like information processing proteins that have hundreds of paralogous examples per genome). Further exploring the differences between the performance of NETL and SNOB for rare and common functions separately is likely to result in improved performance via hybrid methodologies.

In conclusion, we have presented a significant step forward in the calculation of negative examples for protein function prediction. Following the example set for negative protein-protein interactions by the Negetome database [21], we have made our predictions readily available for a variety of organisms. Our NoGO database also includes useful statistics to allow researchers to choose the number of desired negative examples and the likely false negative rate of those examples when used in their own experiments and algorithms.

## Methods and Materials

### Data Processing

Data for the human genome was obtained from the GO database archive, with training annotations obtained from October 2010 and validation annotations from October 2012. The set of genes was obtained from HUGO by selecting all protein-coding gene symbols, resulting 19060 genes. GO terms for these genes were gathered by querying all official symbols for all annotations that have at least one annotated protein in the human genome, resulting in 7432 biological process categories, 2681 molecular function categories, and 997 cellular component categories. GO terms are fully propagated according to the “True Path Rule”, meaning that an annotation of a protein to a particular term also implies annotations to all ancestral terms.

For the RNA Binding term example, there were 686 positive annotations (including IEA) in our training data, and with an additional 157 annotations added in temporal holdout validation data. To these 157 new annotations, we added an additional 381 annotations, which were obtained from [16], but are not yet present in GO. This raised the total of potential false negatives to 538.

For the case study in *Arabidopsis Thaliana*, all data was obtained from the supplementary materials provided by [14].

Annotation data for the GO:0007005 golden set in yeast was obtained from [17], with training GO annotations obtained from the GO ontology in April 2013. The yeast annotations were taken for the same set of genes as the original positive and negative classes defined in [17], comprised of 4966 unique yeast gene symbols, with annotations in 4226 biological process categories, 2231 molecular function categories, and 820 cellular component categories.

Data for the NoGO database was obtained from GO for each organism, with training data for the negative examples collected in April 2013, and training data for the validation plots collected in October 2011 and validated with the April 2013 data. The gene sets for each organism were also obtained from GO, by extracting all unique official gene symbols within that organism which had at least one annotation in any branch of GO. Table 2 lists the number of genes and GO categories for each organism, as well as the NCBI Taxa ID for each specific species used.

**Table 2:** Gene counts, GO term category counts, and NCBI_Taxa IDs for each of the organisms in the NoGO database.

Organism	NCBI Taxa ID	Genes	BP Categories	MF Categories	CC Categories
Arabidopsis	3702	30266	3074	2338	577
Yeast	4932	6380	3533	2091	756
Mouse	10090	25488	9340	3284	1127
Human	9606	18851	9885	3732	1238
Rice	39947	58747	3115	1988	534
Worm	6239	16154	3074	1476	596

### Validation Plot Generation

In order to generate the validation plots in Figure 1 and Figure S1, we plot the average number of false negatives as a function of the number of negative examples. For algorithms that allow the specification of the size of the negative class, we sample the number of false negatives at 100, 200, 500, 1000, 2000, and 3000 negative examples. The average number of false negatives is determined using the temporal holdout, by seeing how many proteins that were designated as negative received an annotation in the function in question (including an IEA annotation). Functional categories that received no new annotations during the temporal holdout are not evaluated, nor are categories with fewer than 3 or more than 300 annotations. Plots are broken down by branch of the GO hierarchy, with each plot showing an average of the results for functions in that branch that meet the specified criteria. The plot for Figure 2 is identical in construction, but for one specific GO category, rather than an average over GO categories.

The plots in Figure 5 and Figure S2 are three representations of algorithmic performance on all organisms in the NoGO database, and each organism, respectively. The leftmost graph was generated by sampling the number of false negatives at negative class sizes equal to 0.1%, 0.5%, 1%, 2.5%, 5%, 10%, 15% and 20% of the size of the genome of the organism in question. This value is then turned into a single number by computing the area under the sample curve for each algorithm, and for the random baseline. These numbers are summed over all categories in the organism (or in the case of Figure 5 across all categories in all organisms), and then divided by the number obtained from the random baseline. The central graph is calculated identically, except here the area under the curve for each algorithm is divided by the random baseline area *before* being summed over all categories, meaning that each GO category contributes equally to the score, regardless of the number of annotations in that category. The rightmost graph represents the total false negative rate, over all GO categories in each branch, when predicting a number of negative examples equal to the number of positive annotations for that GO category. All false negative statics are obtained via a temporal holdout.

Note that in the plots for performance in the NoGO database, it is possible for algorithms to appear worse than the random baseline. This is due to the fact that the random baseline chooses from all possible unlabeled proteins, whereas the algorithms are constrained to only those proteins with GO annotations. Since it can often be the case that new annotations in the temporal holdout set are concentrated among proteins that are already partially annotated, the GO-restricted algorithms are penalized over the random baseline.

### Selection of Negatives through Observed Bias (SNOB) Implementation

The Selection of Negatives through Observed Bias algorithm takes as its basis the pairwise conditional probability calculation of seeing annotation *a* given the presence of annotation *m*, which is specified for the ALBias algorithm in Youngs et al., 2013:


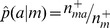
, where 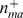 is the number of genes where *m* appears alongside *g* in the dataset, and 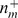
is the total number of genes annotated with *m* in the dataset. As mentioned in the results, SNOB removes the restriction that the score is calculated from leaf annotations only, or that a protein must have an annotation in the same branch as the GO term in question in order to be chosen as a negative. In addition, all annotations are utilized, including IEA annotations. The score vector 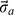, which holds the scores for all genes as potential negative examples for a given GO function *a*, is calculated as the average of the conditional probabilities of all other annotations in each gene, which is efficiently calculable as: 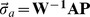, where **A** is the annotation matrix of the dataset, with each row representing a gene and each column a GO category, **W** is the diagonal matrix with **W**_ii_ equal to the total number of annotations for protein i, and **P** is the conditional probability matrix with 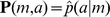. These scores are then ranked to produce a list of negative examples, with the lower scores indicating higher probability that a particular protein is a negative example for the GO term in question.

### Negative Examples from Topic Likelihood (NETL) Implementation

For the Negative Example from Topic Likelihood algorithm, we again formulate a protein as a document, with GO annotations (including IEA) from all three branches as the words in that document. We then run Latent Dirichlet Allocation (Code obtained from David Blei's “lda-c” package) on the document corpus to identify the parameters of the Dirichlet topic distribution, and perform inference on each document to obtain the posterior topic distribution given the GO terms present in that protein (See [18] for the details of LDA). Ideally, we would set the number of latent topics *t* equal to the number of GO categories *m*, but this choice yields infinite perplexity in the corpus, as the number of unique words *w = m* as well. In order to achieve *w* >> *t*, to increase the quality of the learned topics, yet also to preserve coverage of all GO categories, we set the number of topics for each organism equal to the total number of annotated direct descendants of the root ontology terms. For example, in our Human validation data, the biological process node has 27 direct descendants with annotations in the data, the molecular function node has 14 direct descendants, and the cellular component node has 10, for a total of 51 latent topics. By invoking the inverse of the true path rule, whereby negative examples are propagated downwards through the GO graph, this approach guarantees coverage of all GO categories for the purposes of negative example selection.

Since LDA discovers latent topics, which are not predefined before the algorithm is run, it is not immediately obvious which learned topic corresponds to which GO term. Indeed despite our efforts to ensure coverage of every GO category directly descended from a root node, it is not necessarily the case that the correspondence between the topics and the selected GO terms are 1–1. Instead it is possible, even likely, that some combinations of topics/GO terms relate to each other, making exact inference of the probability that a given protein possesses a given GO term difficult under the LDA model. To overcome this problem, we chose to represent the positive class with the average of the Dirichlet posterior vectors for all proteins annotated to the function in question (including IEA annotations). Then for each unlabeled protein *u*, we calculate a Distributional-Overlap Score (DOS) representing the similarity of topics distributions between *u* and the positive class average topic distribution. This score can be viewed as a symmetric simplification of the Kullback-Leibler Divergence metric, and is calculated simply as 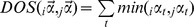, where 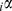 and 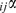 are two Dirichlet posterior parameter vectors (since each posterior vector sums to 1, the DOS score is also bounded by [0,1]). The unlabeled proteins are then ranked according to this score, with the lowest DOS values indicating the most likely negative proteins, as these are proteins which are least likely to share topics with the positive class of proteins.

### Random Baseline Implementation

In order to calculate the random baseline, we consider the positive class to be all proteins with an annotation in the function of interest (including an IEA annotation), and all other proteins to be the unlabeled class. We sample uniformly at random without replacement from those unlabeled proteins in order to pick negative examples, allowing the user to specify the desired size of the negative class. In order to reduce noise from this stochastic operation, we calculate the baseline 100 times for each branch of GO, and then display the average of those 100 calculations.

### Rocchio Implementation

In order to adapt the Rocchio algorithm to protein function, we follow the pseudocode in [12], treating the set of GO terms across all three branches as our lexicography, each protein as a document, and the annotations of that protein as a word. This formulation allows the computation of the *tf-idf* vectors required by the algorithm, and for each function we treat the positive class as all proteins with an annotation in that function (including IEA), and the rest of the proteins as the unlabeled class. The algorithm then builds a representative vector for the positive and unlabeled class, and computes the cosine similarity of the *tf-idf* vector for each unlabeled protein with each of the representative vectors. Where the traditional algorithm would assign as negative examples all proteins whose similarity to the unlabeled class vector is greater than to the positive class vector, we assign a score to each protein, defined as: UnlabeledSimilarity – PositiveSimilarity. This allows us to rank the proteins in terms of confidence of their negativity, with the highest-scoring proteins as the most likely to be negative examples.

### 1-DNF Implementation

For the 1-DNF algorithm, we again formulate proteins as documents and GO terms across all three branches as words. We proceed according to the pseudocode laid out in [4], utilizing as the positive class all proteins with an annotation in the function of interest (including IEA). Other GO terms that appear more frequently in the positive set than the unlabeled set are considered our “enriched” words, and negative examples are all proteins that are not in the positive class and do not contain any of these enriched words. As there is no immediately obvious way to translate this decision into a score, we only implemented this algorithm for one choice of the number of negative examples, rather than thresholding it to allow the user to specify the desired size of the negative class.

### AGPS Implementation

Code for the AGPS algorithm was generously provided by the authors of [7]. AGPS requires features to operate, which we obtained through the similarity networks provided by the Genemania server [22]. Each of these networks (235 networks for human, 297 for yeast) represents similarity between pairs of genes according to a particular datatype. For human data it was necessary to translate the networks from being specified by ENSEMBL ids to gene symbols by using the HUGO lookup for gene symbol and ENSEMBL pairs. For both yeast and human, we performed a simple linear combination of all of the networks, where each component network and the final network was normalized according to the scheme: 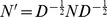, where D is the diagonal row sum matrix of W. Once the final network was obtained (a 19060×19060 matrix for human, 4966×4966 for yeast), we applied Principal Component Analysis to reduce the feature size to a 19060×200 matrix and a 4966×200 matrix, which were the input feature sets for AGPS for each organism, respectively. We ran the algorithm provided by the authors using all of the default constants provided, but as described in the author's text, ran cross-validation for each category and only used negative examples that were chosen in the majority of the cross validation runs. We choose to segment data into 5 cross-validation segments.

AGPS was only validated on functional categories with at least 85 annotations (the reliance of the method on cross-validation increases the number of necessary positive examples for a meaningful result). The lengthy runtime of the algorithm also restricted our application of the method to function categories with more than 85 annotations. To allow for a fair comparison to other methods we utilized the inverse of the true path rule, and for GO functions with fewer than 85 annotations in the human genome, we set the negative examples as the union of all of the negative examples of all parent categories of that GO term.

### Sibling Heuristic Implementation

For the heuristic that chooses siblings as negatives for a function, we follow the specification laid out in [11], whereby a protein is a negative for a function if it is annotated to the parent of that function, but not to the function itself. This includes proteins annotated to sibling categories, as well as those annotated to the parent but to none of the children of that parent. Because some function categories will have no proteins that satisfy these requirements, we revert in this case to the strategy of choosing all non-positive proteins as negative, where the positive class is all proteins with an annotation in the function in question (not including IEA annotations). As Mostafavi 2009 points out, the sibling approach is problematic in that many sibling categories are not mutually exclusive, but we present the technique here for completeness. Since the heuristic will produce different numbers of negative examples for different function categories, the point on the validation plot corresponding to this algorithm represents an average over different sizes of the negative class.

### Function Prediction Implementation

For function prediction, we used our previously published algorithm [5]. Training GO annotations were obtained from the GO archive in April 2013, with validation annotations obtained in December 2013. Input data included protein-protein interaction, Interpro database data [23], gene expression data, sequence similarity, and phylogenetic profiles. Predictions were made for all terms in all three branches, regardless of specificity, but validations were calculated only for those terms that received new annotations during the temporal holdout period.

For each term predicted, the number of negative examples was selected to be the maximum of the number of positive examples of that term, or 20% of the size of the genome. A further restriction capped the number of negative examples at 50% of the number of non-positive genes for the function in question. The area under the curve of the Receiver Operator Characteristic plot was calculated using the methodology presented in [5].

### Data Access

Negative examples are available in the NoGO database, located at: bonneaulab.bio.nyu.edu/nogo.html. Negative examples are currently available for the following species: Human, Mouse, Yeast, Rice, Arabidopsis and Worm. For each function in each organism, a ranked list of genes shows the most to least likely negative examples, available for the SNOB, NETL, and Rocchio algorithms described here. All negative examples were computed using GO data from April 2013.

Accompanying each list is a validation plot (See Figure 2 for a sample, GO:0003723 in Homo Sapiens), which shows the performance of SNOB against a random baseline, trained on GO data obtained from October 2012 and validated with data from April 2013. This plot gives a researcher an idea of the relative performance of the SNOB algorithm against the random reference, in order to give confidence as to the likelihood of false negatives, and also allows a researcher insight into how many negative examples to choose based on the false negative rate presented in the graph.

MATLAB code for generating negative examples from custom data is also be available from the downloads section of the NoGO database, as well as directly from: http://markula.bio.nyu.edu:8080/downloads. The database will be updated with negative examples computed from new GO annotations in April 2014, and then subsequently every three months.

## Supporting Information

Figure S1 **Specificity-segmented performance.** Performance of negative example selection algorithms broken down by specificity for **a.** Biological process, **b.** Molecular Function and **c.** Cellular component. Specificity is defined by the number of annotations present for a GO category in the human genome training data, split into buckets of size: 101–300, 31–100, 11–30, and 3–10.
(TIF)

Figure S2 **Performance metrics broken down by organism.** Organism plots for a) Arabidopsis, b) Yeast, c) Mouse, d) Human, e) Rice, and f) Worm. The leftmost graph for each organism represents the average area under the false negative curve, as a percentage of the area under the random baseline curve, weighted by the number of annotations in each GO category. The central graph is the same set of values re-calculated so that each GO category contributes equally to the average, regardless of specificity. The rightmost graph depicts the false negative rate for each algorithm when predicting the same number of negative examples as the number of positive annotations for each GO category.
(TIF)

Figure S3 **Performance measures including the sibling method.** These plots are duplicates of the performance plots in Figure 1 of the paper, but including the Sibling Negatives heuristic, to illustrate the poor performance of that heuristic.
(TIF)

Figure S4 **Performance measures evaluated without IEA annotations.** Performance measures for negative example prediction on the human genome, in each of the three branches of GO. These results are the similar as those presented in Figure 1, with the difference being that here error rates are calculated using only curated GO annotations, and ignoring IEA annotations.
(TIF)

Figure S5 **Specificity-segmented performance evaluated without IEA annotations.** Performance of negative example selection algorithms broken down by specificity for **a.** Biological process, **b.** Molecular Function and **c.** Cellular component. Specificity is defined by the number of annotations present for a GO category in the human genome training data, split into buckets of size: 101–300, 31–100, 11–30, and 3–10. These results are similar to those presented in Figure S1, with the difference being that here error rates are calculated using only curated GO annotations, and ignoring IEA annotations.
(TIF)
