# Supplementary figures and images for: Negative Example Selection for Protein Function Prediction: The NoGO Database
Source: PLoS Comput Biol. 2014 Jun 12;10(6):e1003644. doi: 10.1371/journal.pcbi.1003644 (PMC4055410; doi:10.1371/journal.pcbi.1003644)

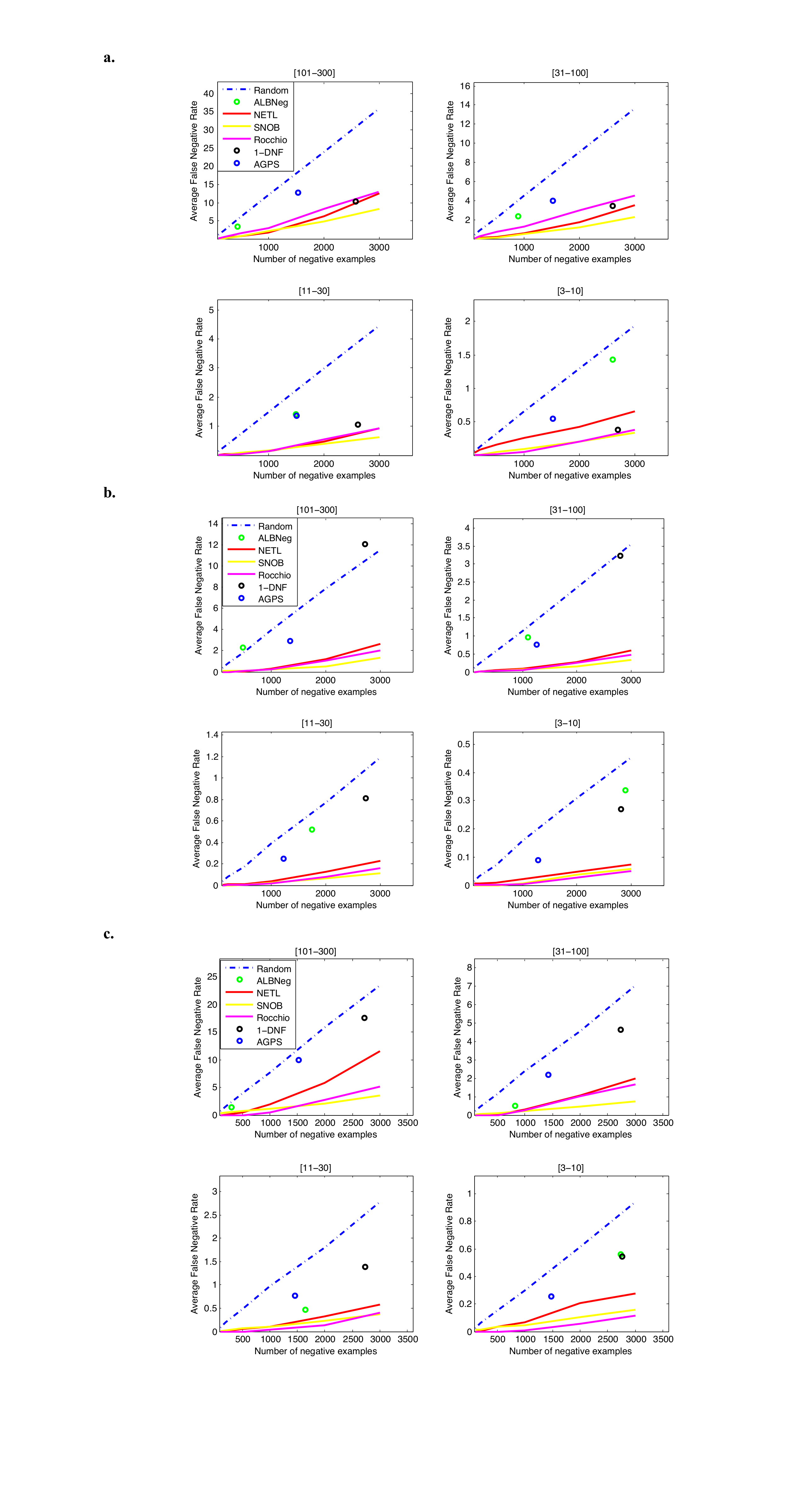

Supplement: Figure S1 — Specificity-segmented performance. Performance of negative example selection algorithms broken down by specificity for a. Biological process, b. Molecular Function and c. Cellular component. Specificity is defined by the number of annotations present for a GO category in the human genome training data, split into buckets of size: 101–300, 31–100, 11–30, and 3–10. (TIF) [file pcbi.1003644.s001.tif]

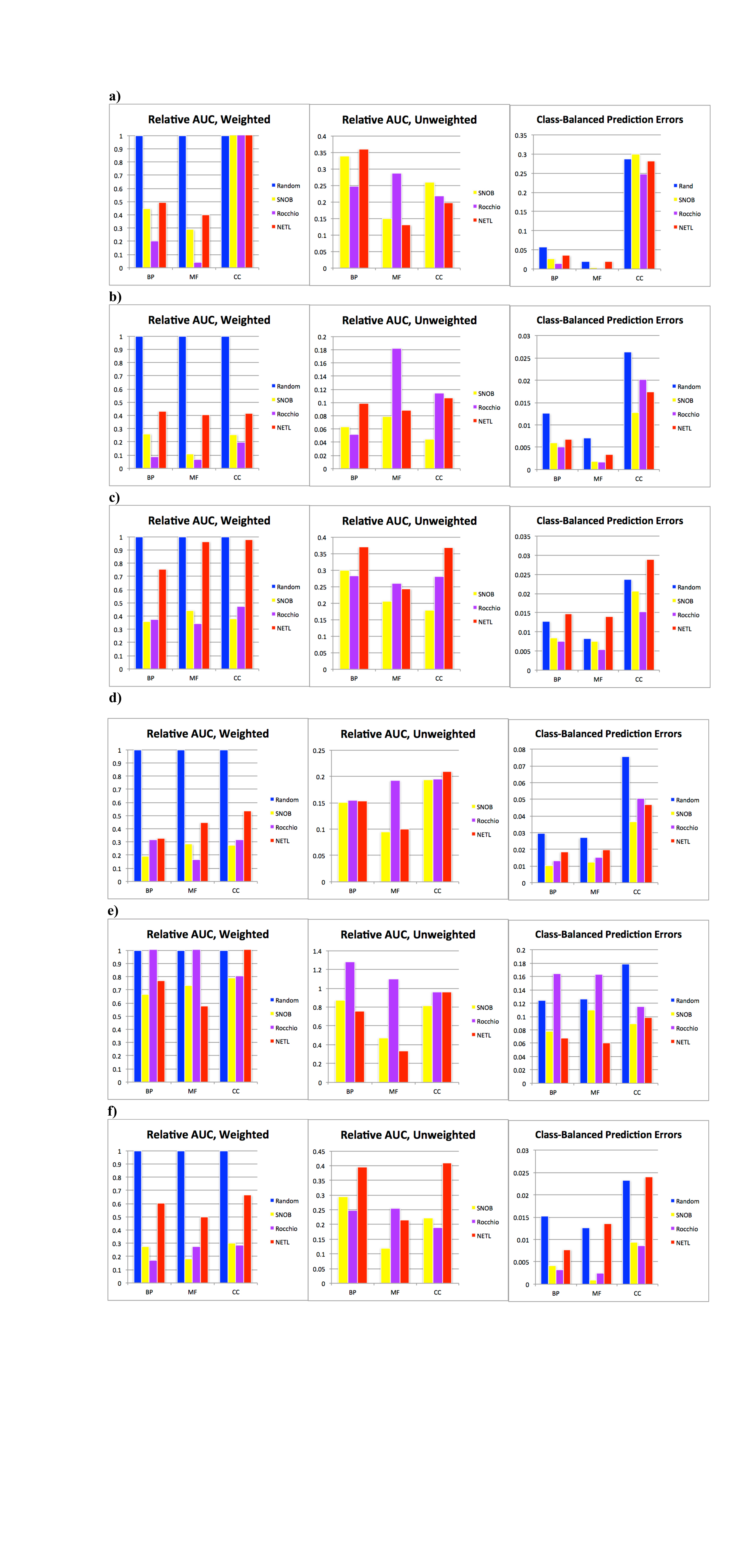

Supplement: Figure S2 — Performance metrics broken down by organism. Organism plots for a) Arabidopsis, b) Yeast, c) Mouse, d) Human, e) Rice, and f) Worm. The leftmost graph for each organism represents the average area under the false negative curve, as a percentage of the area under the random baseline curve, weighted by the number of annotations in each GO category. The central graph is the same set of values re-calculated so that each GO category contributes equally to the average, regardless of specificity. The rightmost graph depicts the false negative rate for each algorithm when predicting the same number of negative examples as the number of positive annotations for each GO category. (TIF) [file pcbi.1003644.s002.tif]

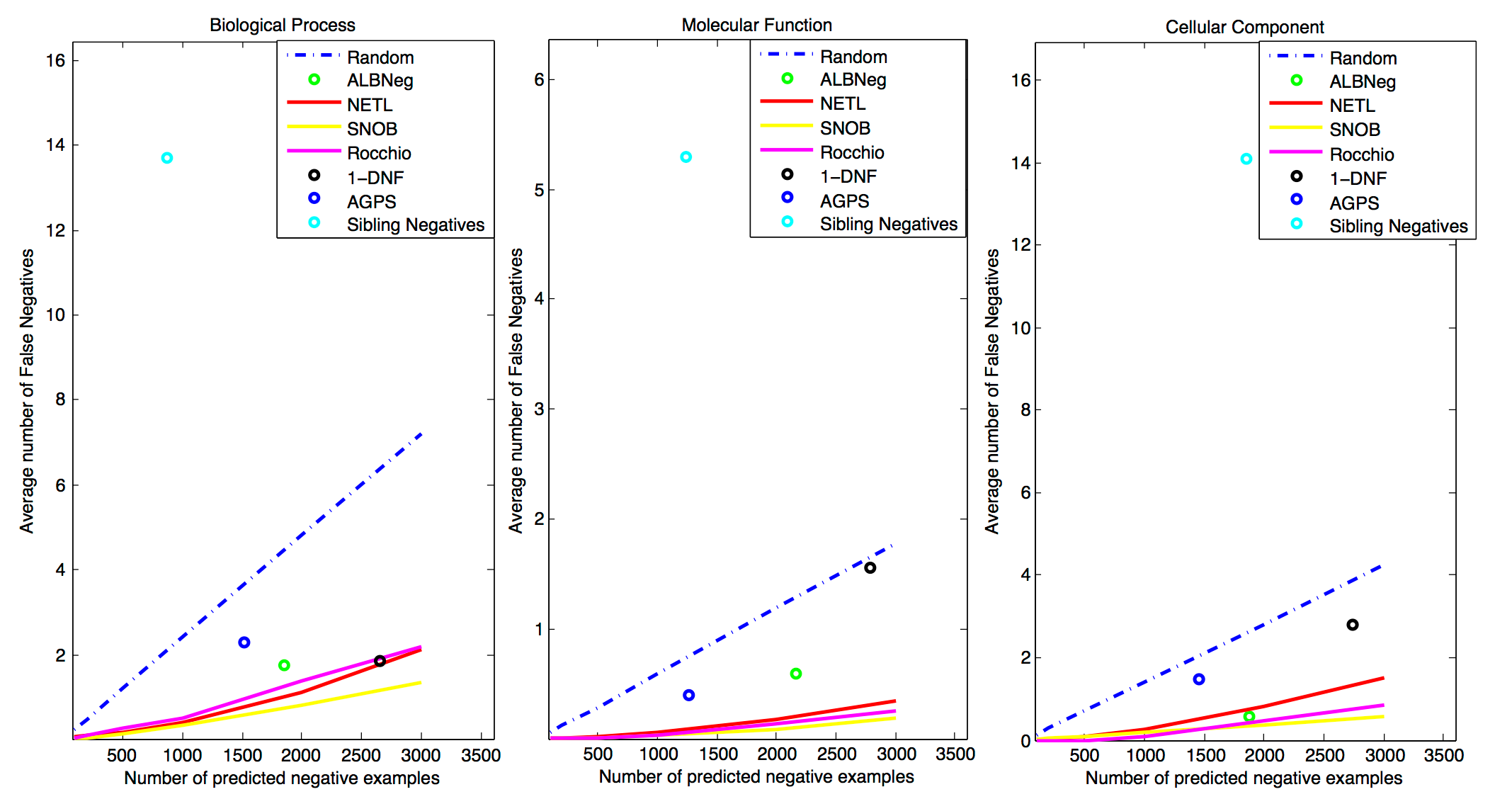

Supplement: Figure S3 — Performance measures including the sibling method. These plots are duplicates of the performance plots in Figure 1 of the paper, but including the Sibling Negatives heuristic, to illustrate the poor performance of that heuristic. (TIF) [file pcbi.1003644.s003.tif]

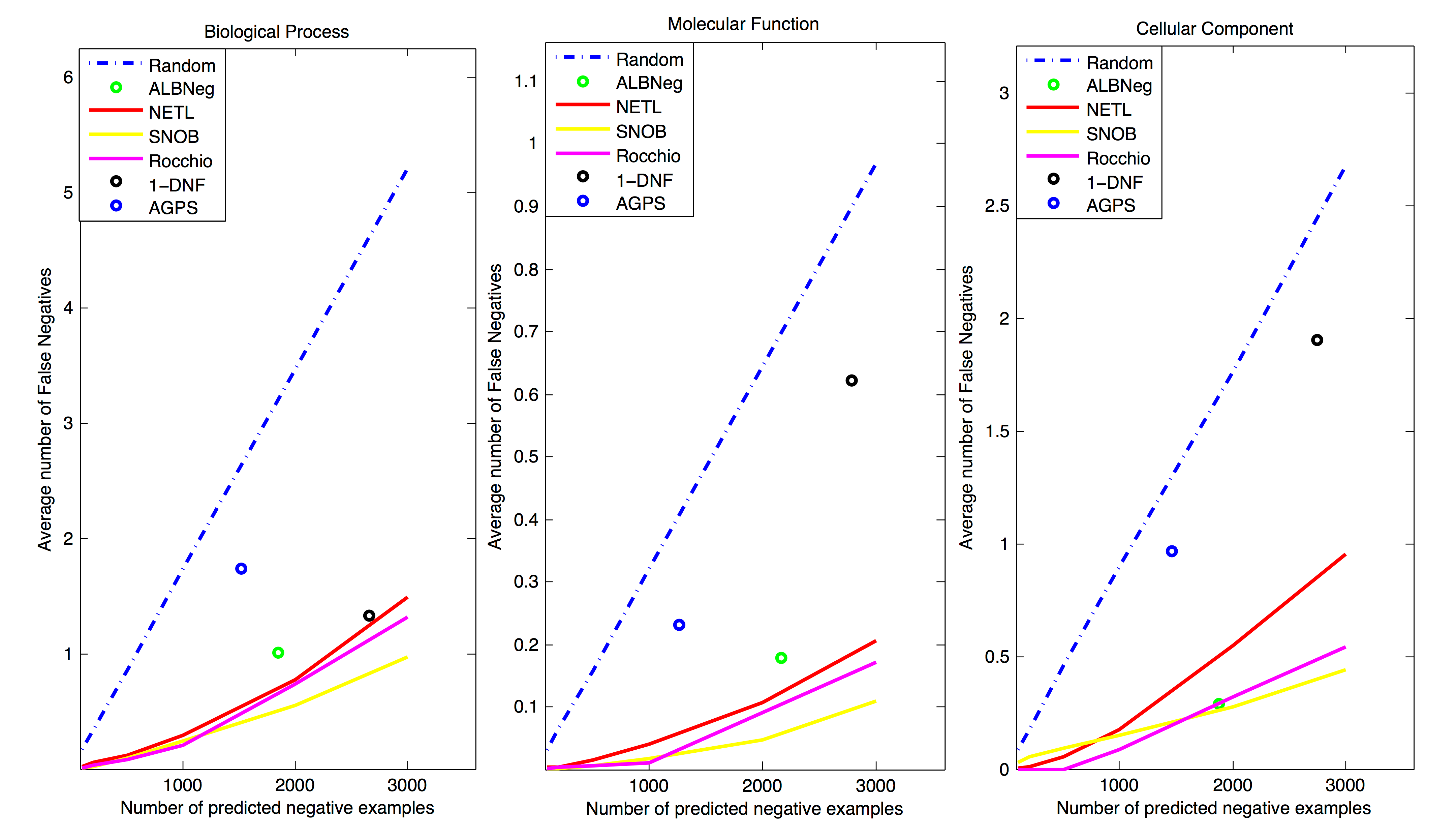

Supplement: Figure S4 — Performance measures evaluated without IEA annotations. Performance measures for negative example prediction on the human genome, in each of the three branches of GO. These results are the similar as those presented in Figure 1, with the difference being that here error rates are calculated using only curated GO annotations, and ignoring IEA annotations. (TIF) [file pcbi.1003644.s004.tif]

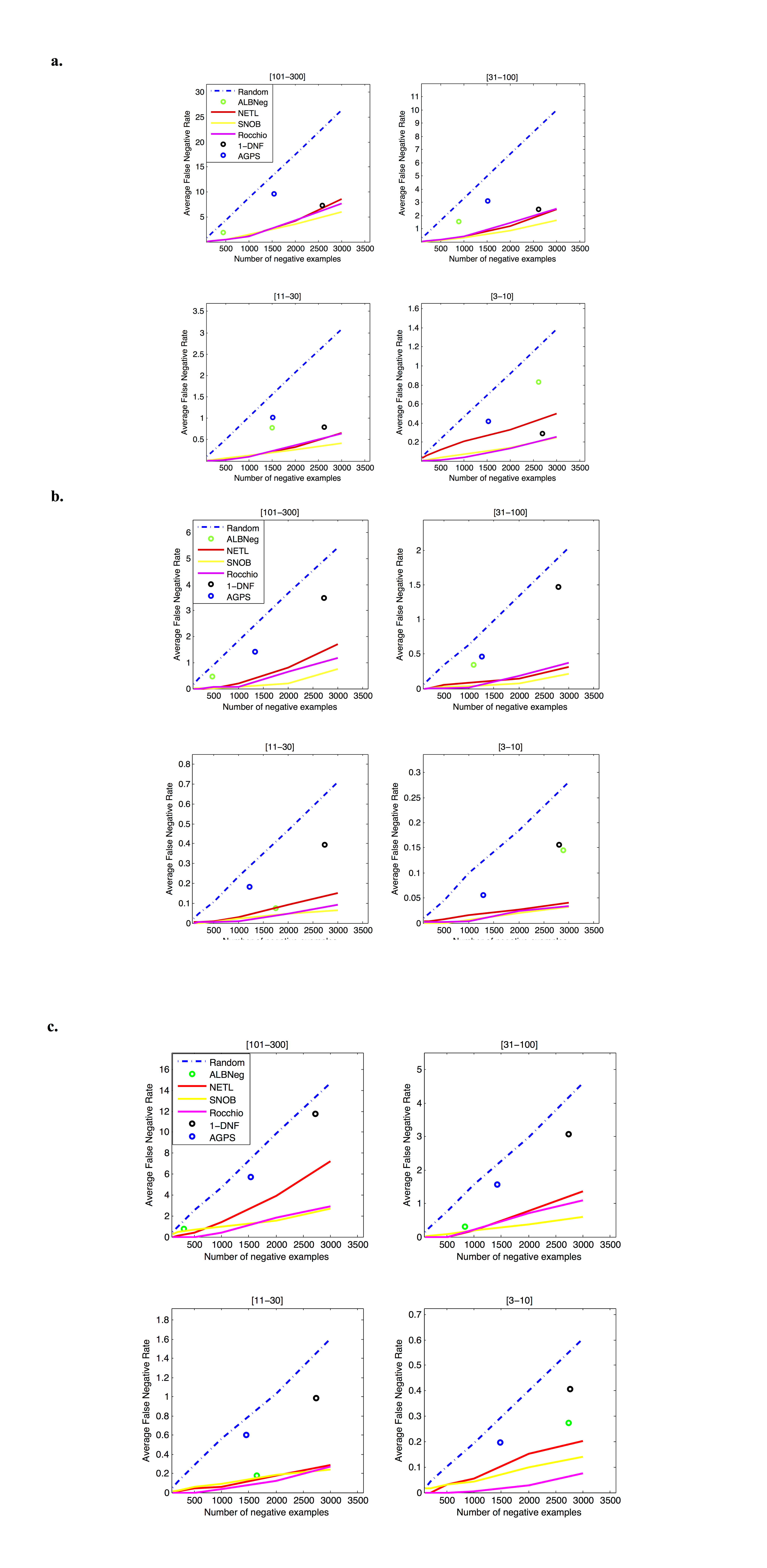

Supplement: Figure S5 — Specificity-segmented performance evaluated without IEA annotations. Performance of negative example selection algorithms broken down by specificity for a. Biological process, b. Molecular Function and c. Cellular component. Specificity is defined by the number of annotations present for a GO category in the human genome training data, split into buckets of size: 101–300, 31–100, 11–30, and 3–10. These results are similar to those presented in Figure S1, with the difference being that here error rates are calculated using only curated GO annotations, and ignoring IEA annotations. (TIF) [file pcbi.1003644.s005.tif]
